# Online learning in proton radiation therapy: the future in the post-Covid-19 pandemic era?

**DOI:** 10.1259/bjro.20210054

**Published:** 2021-12-10

**Authors:** William Croxford, Anna France, Matthew Clarke, Lauren Hewitt, Karen Kirkby, Ranald Mackay, Jane Miller, Ganesh Radhakrishna, Alison Sanneh, Ed Smith, Shermaine Pan

**Affiliations:** The Christie NHS Foundation Trust, Manchester, UK; The University of Manchester, Manchester, UK

## Abstract

**Objective:**

The Covid-19 pandemic placed unprecedented strain on medical education and led to a vast increase in online learning. Subsequently, the Christie International Proton School moved from face-to-face to online. Delegate feedback and current literature were studied to determine benefits, challenges, and potential solutions, for online proton therapy education.

**Methods:**

The course was converted to a 6-week online course with twice weekly 2-h sessions. Feedback was studied pre-, during-, and post-course regarding demographics, learning objectives, proton therapy knowledge, ease of engagement, technical difficulties, and course format. Statistical analyses were performed for proton therapy knowledge pre- and post-course.

**Results:**

An increase in delegate attendance was seen with increased international and multidisciplinary diversity. Learner objectives included treatment planning, clinical applications, physics, and centre development. Average learner reported scores of confidence in proton therapy knowledge improved significantly from 3, some knowledge, to 4, adequate knowledge after the course (*p*<0.0001). There were minimal reported difficulties using the online platform, good reported learner engagement, and shorter twice weekly sessions were reported conducive for learning. Recordings for asynchronous learning addressed time zone difficulties.

**Conclusion:**

The obligatory switch to online platforms has catalysed a paradigm shift towards online learning with delegates reporting educational benefit. We propose solutions to challenges of international online education, and a pedagogical model for online proton therapy education.

**Advances in knowledge:**

Online education is an effective method to teach proton therapy to international audiences. The future of proton education includes a hybrid of online and practical face-to-face learning depending on the level of cognitive skill required.

## Introduction

In March 2020, the WHO declared Covid-19 a global pandemic^1^ after the initial SARS-Coronavirus 2 virus was detected in Wuhan, China in December 2019.^2^ The importance of social distancing in an attempt to control the pandemic has put a strain on the accepted norms of medical education around the world, including the UK. This has led to adaptations of teaching programmes^3,4^ and conferences^5,6^ from an in-person format to a purely online setting, to maintain and share important learning during the pandemic.

Online learning has been present in medical education pre-pandemic, with a predominantly asynchronous approach, *e.g.* conferences providing breaking research updates online after the event,^7^ and the use of online asynchronous cases or modules to teach students in medical schools.^8^ Some limitations to online learning have been previously explored; these include a perceived lack of IT skills, negative attitudes, infrastructure, and high costs.^9^ In response to constrained face-to-face educational options, medical educators were compelled to overcome these challenges. This led to implementation of new online learning platforms and courses being remodelled to online formats, whilst also whetting the appetite of learners to the untapped benefits of online learning, helping change attitude and culture.^10^

The Christie Proton School, a collaboration between the University of Manchester, the Christie Proton Beam Therapy (PBT) Centre and Christie School of Oncology, had previously delivered a face-to-face Proton School. During the pandemic, the second Proton School was designed to be entirely online from November to December 2020 over 6 weeks; this time period spanned the second national UK lockdown, further emphasising the importance of an entirely online programme.

We propose that there are benefits and limitations for teaching in a solely online format based on our experience of delivering an international multidisciplinary online educational programme on proton therapy.

## Methods and materials

The Christie Proton School had been delivered once before as a 5 day face-to-face course with registering attendees from the UK and Australia. It was delivered over 1 week with a focus on physics, radiobiology, service and centre development, and multidisciplinary teaching on the treatment of head and neck, brain, base of skull, paediatric and sarcoma cancers with proton therapy.

The Christie Proton School was converted to an online format during the Covid-19 pandemic. A digital provider supplying a teaching and learning customised online platform was used. The course was delivered from 4 November to 9 December 2020 synchronously with two 2-h sessions a week from 08.00 to 10.00 GMT on different days. Attendees had the opportunity to engage with the panel of educators and ask questions throughout sessions via a live chat box. Questions were then answered and debated amongst educators in a short question and answer (QA) session at the end. To ensure smooth running of the sessions, chairs and multidisciplinary teachers (clinicians, radiographers, physicists, engineers and university lecturers) were trained to use the online platform before the event.

Feedback forms were sent by email to attendees to complete prior to the event to determine demographics, baseline knowledge of PBT and learning outcomes. Specific feedback on each session was collected on each day to determine educational value, engagement and ease of using the online platform. Feedback at the end of the course also assessed value for money, administration, course scheduling, progression of PBT knowledge and familiarity, whether learning outcomes were met, and whether they would recommend the course. Both quantitative and qualitative data were collected. There was a mixture of Yes/No, 5-point Likert scale (*e.g.* no familiarity, 1, to extremely familiar, 5, with PBT) and free text questions.

Score data are described using summary statistics, and visualised using bar charts and balloon plots. Qualitative data underwent thematic content analysis using an inductive approach, with initial open coding before themes were collected, summarised and overlapping themes removed. Larger qualitative data sets, such as delegates’ intended learning outcomes, were peer reviewed independently by a second researcher for verification of the identified themes. Pre-course perceived proton knowledge was scored by delegates on both the pre- and post-course questionnaires, with delegates also scoring their post-course knowledge on the post-course questionnaire. This enabled use of both unpaired and paired statistical testing to assess if there was significant change between pre- and post-course perceived knowledge scores, aiding in the assessment of whether candidate educational needs were met. A χ^2^ test was used to assess for an association between pre- or post-course questionnaire and knowledge scores, carrying out a correlation approach to assess the impact of the course on delegates’ perceived knowledge.^11^ Further, a permutation test was used to assess differences in the paired knowledge scores on the post-course questionnaire. Significance was assessed at the 5% level.

## Results

### Demographics

Attendance increased from 6, at the first face-to-face course pre-pandemic, to 103 delegates at the online course from a number of different international countries including, the UK, Argentina, Australia, Belgium, Canada, Germany, Greece, Italy, the Netherlands, Norway, Saudi Arabia and Sweden.

**Figure 1.**
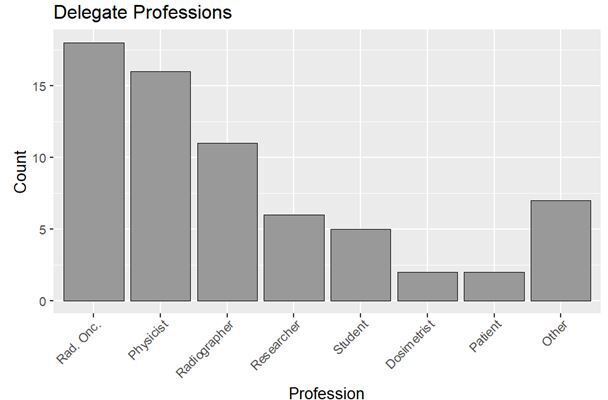
Bar chart showing delegate profession.

68 (66%) of delegates answered the pre-course questionnaire. This demonstrated that the majority of responders, 45 (66.2%) delegates, were clinicians, physicists or radiographers (Figure 1). ‘Other’ attendees, shown in Figure 1, included engineers, a quality manager, government dosimetry auditor, statistician, lecturer and trial coordinator.

58 (56.3%) completed a post-course questionnaire.

### Learning objectives and outcomes

Learning priorities for delegates were determined on the pre-course questionnaire by asking the free text question, ‘what would you like to gain from the course?’ There were 59 free text responses and delegates often had more than one learning outcome. Common learning outcome themes were identified using thematic content analysis. Table A in Supplementary Material 1 demonstrates the process of our analysis, with highlighted phrases from feedback included within each identified theme. Table 1 shows the identified learning outcome themes and how frequently they were written by delegates.

**Table 1.** Table showing frequency of identified learning outcome themes written by delegates

Learning outcome	Number of delegates, N (%)
Overview of proton therapy knowledge	22 (37.3%)
Treatment planning	14 (23.7%)
Clinical application	13 (23.0%)
Proton therapy physics	12 (20.3%)
Centre development	10 (16.9%)
Treatment preparation and delivery	8 (13.6%)
Clinical trials and research	7 (11.9%)
Develop a proton network	2 (3.4%)

**Figure 2.**
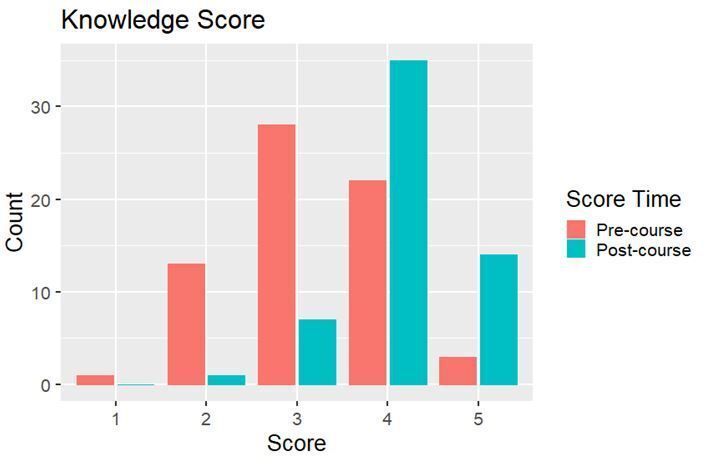
Bar chart illustrating pre- and post-course PBT perceived knowledge scores (one being no knowledge and five being extensive knowledge). PBT, proton beam therapy.

Using attendees scoring of their perceived proton knowledge (Figure 2), a χ^2^ test was performed to assess whether there was a statistically significant difference between the unpaired pre-course questionnaire score and the post-course score of knowledge. This gave a χ^2^ test statistic of 82.8 (df = 4), and a *p*-value of less than 0.0001, showing there is a highly statistically significant association between delegates’ perceived knowledge score and whether delegates were scoring their knowledge pre- or post-course. To more thoroughly assess the impact of the course on perceived knowledge, Pearson’s correlation coefficient was calculated. This gave a correlation coefficient of 0.754 (*p* < 0.0001), indicating perceived knowledge of proton therapy was significantly increased by course attendance. A permutation test was carried out to test for a significant difference between the paired pre- and post-course knowledge scores provided by delegates on the post-course questionnaire, resulting in a *p*-value less than 0.0001. This again shows a highly significant difference in the pre- and post-course perceived knowledge scores. With the median pre-course score being 3, and a median post-course score of 4, we can conclude that the course was successful in significantly increasing attendees’ confidence in their knowledge of proton therapy. In addition, 97.4% of responding delegates answered yes to their learning outcomes being met on the post-course questionnaire.

### Learning environment and experience

**Figure 3.**
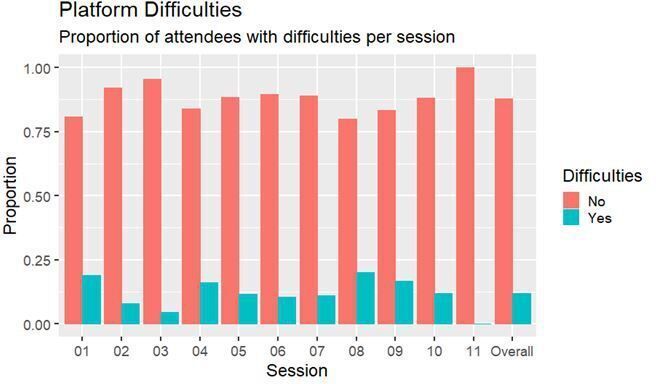
Bar chart showing proportion of delegates who had difficulties with the online platform per session and on average overall.

Out of the 103 registered delegates, a median of 2 reported having difficulties with the online platform per session. The maximum was 8, which was in the first session, and in Session 11, zero delegates reported any difficulties. Of the delegates who answered the individual session feedback, the percentage of delegates reporting platform difficulties varied from 0 to 20% throughout the course, with an average of 12% per session (Figure 3).

Qualitative platform difficulty feedback was analysed in individual session and post-course questionnaires using thematic content analysis. Delegates reported more difficulties in sessions 1 and 8, and this was reflected in the qualitative data of those who said ‘yes’ to experiencing platform difficulties; with six and three questionnaire feedback comments respectively. Two themes were identified in Session 1; audiovisual (AV) and early setup problems. One theme was identified in Session 8; AV problems. However, positive comments were seen in all individual and post-course feedback regarding “little technical difficulties”. See Supplementary Material 1 for qualitative feedback from delegates. Feedback emphasises the importance of trial logins with appropriate equipment for educators and learners.

It is important to note that individual session feedback had increased rates of non-response; median response rate 18.4% (range 6.8–40.8%). Results should be interpreted with caution but suggest delegates experienced minimal difficulty using the online platform.

**Figure 4.**
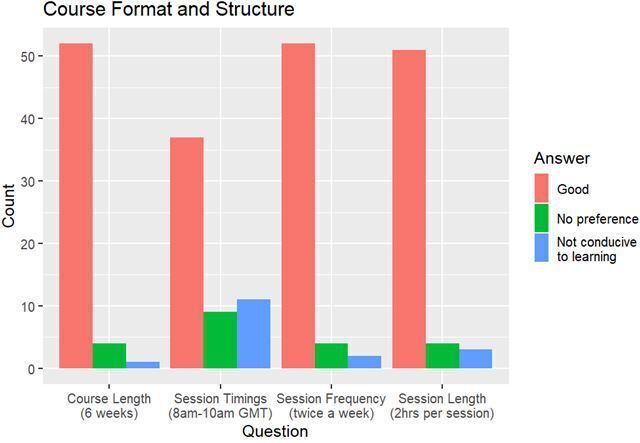
Bar chart showing delegate feedback on course structure and format.

The vast majority of delegates stated the 6 week course duration (91.2%), twice weekly sessions (89.7%) and 2-h length sessions (87.9%) were good for learning (Figure 4). Delegates did not provide qualitative feedback regarding 6 week course duration or twice weekly sessions. However, delegates did comment on the 2-h session length when it was reported to be conducive to learning. These were classified in to a theme: shorter time durations enable better learning/concentration. Some delegates felt sessions were too long and this was related to sessions which ran overtime; request for a break in such circumstances was written by a delegate. See Supplementary Material 1 for qualitative feedback from delegates. Feedback highlights issues around screen fatigue in online learning and the importance of regular breaks.

A smaller majority (64.9%) said the timing of 08.00–10.00 GMT was good, with 19.3% reporting it was not conducive to learning. In the qualitative feedback of those delegates who stated it was not conducive to learning, two themes were identified: difficulty in early morning concentration (UK delegates), and time zone difficulties for family commitments (particularly Australia). See Supplementary Material 1 for qualitative feedback. However, there was positive feedback regarding the value of recordings, *e.g.* “providing recordings of each seminar were especially helpful”, and service provision, *e.g*. timings “were great for the service I have to provide.” Some delegates commented that “organising a time that suited all time zones is very difficult” and that “not being jet-lagged when attending has been excellent.” This suggests that delegates appreciated that the course accommodated an international audience. Feedback highlights the importance of recordings for asynchronous learning, careful consideration by educators of different time zones, and focus on maintaining delegate motivation for online learning at all times.

### Learner engagement with faculty

**Figure 5.**
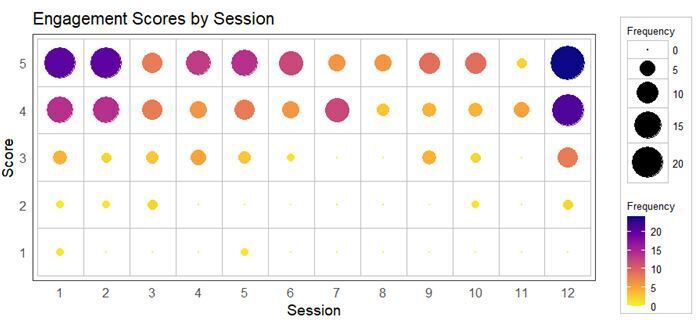
Balloon plot showing delegate feedback on faculty engagement for each session, where the area of each dot is proportional to the amount of delegates scoring that level of engagement within each session.

Faculty engagement was scored on a scale of 1 to 5, with 1 being unable to engage and 5 being extremely engaging. The median faculty score of engagement across all sessions was 4, with the median being 4 or above for each individual session. Figure 5 indicates the frequency of delegates scoring engagement at each of the levels, 1 to 5, for each of the individual sessions. Figure 5 shows the majority of delegates gave an engagement score of 4 or above for each session, with a score of 5 being the most common across all except the 7th and 11th sessions. Half of the sessions have no delegates scoring engagement less than 3. Scores of 2 or below were given by very few delegates, at most two per session. Overall, this demonstrates good engagement between the faculty and delegates.

Two themes were identified from qualitative feedback, including: QA sessions were good for engagement, and some candidates want longer QA sessions. See Supplementary Material 1 for qualitative feedback. One feedback comment in Session 4 stated “I should have participated live.” This could suggest the delegate wanted to ask questions, highlighting the importance of educators sharing contact details at the end of online teaching.

## Discussion

With the conflicting interests of social distancing to contain the Covid-19 pandemic and importance of continuing medical education in post-graduate education, online lectures, webinars and courses have become the norm in this current season. This has presented both opportunities and challenges, highlighted by our online Proton School.

### Opportunities and challenges

For the Christie Proton School, an online programme significantly increased attendance in numbers and created the opportunity for a wider international audience. Waghmare and Gupta^12^ suggest that international faculty are more easily available with online learning and the ease of access, from home or the workplace, and eliminated travel cost, are likely to be contributing factors to the increased attendance at online events. Delegate attendance may have also risen due to an increase in the number of patients treated with proton therapy in the UK and internationally; a second proton centre is due to open in the UK and numbers of international proton centres are rising rapidly.^13,14^

Online learning can be associated with technical challenges,^9,15^ particularly when delegates may not have the IT skills to rapidly learn how to use new online platforms. Our quantitative results suggest that delegates experiencing difficulty using the online platform was minimal, and that difficulties were overcome during the course. Qualitative feedback from Session 1 regarding IT setup issues were no longer present in later sessions. This all suggests that the obligatory engagement with online learning during the Covid-19 pandemic has increased willingness to use online platforms^16^ and that delegates can become more familiar with a particular platform as a course proceeds. It is important that live technical support was available during the online course for those who had platform difficulties as technical issues can reduce intrinsic delegate motivation to continue with an online course.^17^ With 98.3% of learners recommending this course to colleagues in the future, we predict learners will continue to embrace the online environment for professional development beyond the pandemic.

The online learning environment can have difficulties associated with communication and rapport.^18^ However, the median score for faculty engagement was always 4 (good) or above for each teaching session in the online Christie Proton School. Along with positive qualitative feedback regarding QA sessions, this suggests the opportunity for delegates to ask questions via a live chat box provided good learner engagement and interaction. Time was always allowed after each lecture for these questions to be answered, and when interaction was constrained by time, or by those unable to attend live, opportunities to engage with the teaching faculty via email were made available. Motivation for online learning during the Covid-19 pandemic must be carefully considered given stress and anxiety can demotivate learners and can thus negatively affect achievement.^19^ Maintaining delegate attention must be a priority to keep motivation beyond the initial novelty of online learning and good interaction between educator and learner is an important factor.^17,20^ Learner–learner interaction can also help delegates feel they are part of an online community,^21^ preserving motivation for online learning. These factors could explain the numerous positive feedback comments for the interactive QA sessions.

A large majority of participants were in favour of the 2-h length sessions and twice weekly frequency. With the amount of post-graduate online learning exponentially increasing since the pandemic,^22^ there is a risk of screen fatigue and lower concentration levels. Excessive screen time has been shown to affect the sleep cycle, concentration levels and cause fatigue and eye strain.^23^ The shorter session schedule during this online course, rather than whole day sessions over 1 week, helped to minimise screen time, increase concentration levels and can explain the delegates’ preferences in the qualitative feedback.

Importantly, delegates reported a statistically significant increase in confidence in their level of proton knowledge. This demonstrates that online learning can be an effective method to increase delegate perception of proton therapy knowledge.

With a wider international audience, time scheduling for synchronous learning can become difficult in an online school. Qualitative data suggested that some delegates in Australia found evening learning difficult with domestic commitments, and a minority in the UK had difficulty engaging early in the day. However, it was stated by delegates that recordings allowing asynchronous learning at a different time helped them work around domestic or clinical commitments, an important aspect to online learning as emphasised previously by the HeXL NHS e-learning project.^24^ Flexible accessibility to learning via recordings can also have the effect of increasing delegate attendance.^25^ Additionally, lack of international travel and changing of time zones for delegates helps eliminate disruption to circadian rhythms and subsequent jet lag. This has been shown to have deleterious effects on both memory and cognition,^26,27^ thus highlighting a benefit of online learning synchronously during different time zones, with the help of recordings when needed. UK candidates reporting that early morning concentration and learning was difficult again emphasises the importance of keeping delegates motivated to learn online; this could be improved with interaction throughout a session and keeping content highly relevant.^20^

Although a lack of face-to-face networking was a challenge with our online course, this was only directly written twice in delegate feedback as a desired outcome from the course. Online networks and groups using social media are useful ways to share medical ideas among professionals^28,29^ but care needs to be taken for confidentiality. Such groups could be set up for future online proton schools to enable online forum-based discussion. If effective methods for interaction are used, the wider international audience that attends an online course could in fact provide a broader opportunity for networking.

With an increased international audience, there can be problems for attendees from low or middle income countries (LMICs) who lack the infrastructure to engage in these educational activities. This can include issues with hardware, such as lack of suitable computers, or problems related to poor internet connectivity.^30,31^ Effort should be made by higher income countries to help those from LMICs participate where possible, including provision of bursaries.

On analysing the online Christie Proton School quantitative and qualitative feedback, we propose potential solutions for challenges to online learning to optimise the learning experience (Table 2).

**Table 2.** Table showing challenges to online learning and proposed solutions

Challenges	Proposed Solutions
*Technical Difficulties*	Trial log on to online platform prior to event with the same computer/software to be used. Educator to provide information at the start/prior to the session on technical support access.
		*Engaging with the Faculty*	Know how to ask questions; educators to inform how at the start. Educator to maintain learner motivation with interactive elements. Educators to provide email contact for post-course questions for learners. Participation in learner survey to improve future sessions.
*Screen Time Fatigue*	Regular scheduled breaks. Leave your PC during breaks. Walk outside.		
				*Different Time Zones*	Plan ahead: your location and clinical/domestic duties (including applying for study leave if necessary). When needed, access recordings asynchronously. Educator to account for different learner time zones.
*Networking*	Encouragement of all to use chat box when appropriate. Access forum-based discussions on online platforms if available. Consider using secure social media groups – caution regarding confidentiality. Breakout rooms for learners with appropriate facilitator. Possibility of hybrid face-to-face courses post-pandemic.				

### Limitations

Although the feedback data from delegates shows largely positive results for the online Proton School, the pre-course questionnaire was completed by 66% of delegates and the post-course questionnaire by 56% of delegates which could introduce non-response bias. This was a larger problem for the individual feedback sessions where response rate ranged from 6.8 to 40.8%. Questionnaires were available to complete directly after a session on the online platform and email reminders were sent the same day. Responses reduced over time, with the lowest response rate being 6.8% in Session 12, and questionnaire fatigue is likely to have contributed to a rise in non-response.

Non-responders and responders can differ in significant ways,^32^ and non-response bias is a particular challenge in surveys, sometimes making meaningful inferences difficult.^33^ This can be highlighted in the difficulty in accessing the platform section of our questionnaire, where delegates who did not have difficulty accessing may not have felt the need to report, or where those having difficulty accessing IT may not have been able to report. However, it was felt overall, that the median of 2 delegates per session, out of 103 delegates on the course, reporting a difficulty per session represented minimal IT challenges.

Suggestions to minimise non-response bias include keeping surveys short and accessible, and providing incentives for completion.^32^ Incentives can include a certificate of attendance after questionnaire completion, whether sessions are attended live or retrospectively via recordings. Incentives will be considered for the next course, particularly to increase individual session feedback.

On assessing improvement of proton knowledge, this was done before and after the course subjectively with the question, “How would you rate your knowledge of proton therapy and familiarity with this subject matter?” This was not assessed with an objective test before and after the Proton School and was a limitation of our data. A short objective self-assessment will be considered for future Proton Schools.

### Pedagogical Model for an online International Proton School

There are various pedagogical models which can be applied to online learning, *e.g.* the Knowledge, Process, Practice (KPP) model.^34–36^ They describe key elements for delivery of online teaching and discuss how an online programme can be effectively assembled. They discuss the movement towards blended learning, rather than online learning being purely supplementary,^34,35^ and the flexible time and location benefits it can offer post-graduate learners.^36^ The Hyflex model particularly advocates blended learning^37^; this has been increasingly adopted during the Covid-19 pandemic where delegates or students can vary attendance by joining sessions: (1) in person, (2) synchronously online, or (3) asynchronously by recordings. Although this can offer unique challenges to educators regarding student feedback,^38^ it offers flexibility to learners, particularly during the Covid-19 pandemic where intermittent self-isolation is needed.

All online schools should perform equality impact assessments to ensure content is accessible to all. Guidance from the UK government can be found online^39^ regarding assistive technologies and digital accessibility.

We describe seven elements from our experience that are important to consider when creating an online proton school for a post-graduate medical audience. These proposed foundations, LEARNER, include:

- **L** – Learning objectives and assess prior knowledge.
- **E** – Explore and enhance prior understanding, explain new knowledge.
- **A** – Active participation between learners and educators with interactive components.
- **R** – Relaxed and flexible environment for learning. Regular breaks.
- **N** – Networking opportunities with other learners and educators.
- **E** – Electronic, technical and accessibility support available.
- **R** – Reflection/improvement of the course by the educator. Reflection by learners; translating knowledge in to practice.

This model has both similarities and differences with other frameworks for online learning, such as Laurillard’s conversational model^40^ and Salmon’s five stage model for online learning.^41^ Laurillard’s model puts the student at the centre and focuses on interactions between educators and learners, leading to development of concepts through an iterative process. This is reflected in our model by the first three points and networking, where students can learn through acquisition, discussion, enquiry and collaboration. Learning through practice and collaboration could be improved in subsequent courses if candidates are allocated online activities to complete outside lectures, especially in groups. However, the LEARNER model offers a clearer focus on the learning environment, particularly online, with a focus on flexibility, breaks and technical support. Salmon’s five stage model concentrates on the online environment and delivery of education. This includes ensuring technical access is well established early in Stage 1, with an emphasis that online socialisation must also occur early in Stage 2 to maintain learner retention. The LEARNER model also has a stronger online delivery focus, with networking and technical support being key factors, but does not emphasise that these factors achieved early on could improve delegate retention. LEARNER encompasses concepts in both Laurillard’s and Salmon’s model into one holistic approach for online learning.

Putting our pedagogical model in to a wider educational context, it is important to consider where it lies within Bloom’s taxonomy^42^; a well-established series of six hierarchical cognitive levels often used to develop courses. Levels 1 and 2, remembering and understanding respectively, should be achievable for proton education in an entirely online format. This is partly confirmed by delegates reporting improved confidence in their proton therapy knowledge; this would ideally be confirmed by pre- and post-course tests. For levels 3 and 4, applying and analysing, a hybrid format may be more applicable, *e.g.* breakout rooms for smaller supervised online workshop activities supplemented by clinical observerships. Levels 5 and 6, evaluating and creating, requires direct hands on practice in a proton department but elements can still be achieved online or virtually. For example, virtual simulation of patient setup, treatment planning, and plan delivery of a case can be discussed, debated, and visualised, using the Proton VERT programme.^43^ Furthermore, using a flipped classroom approach,^44^ where delegates learn distributed pre-course material prior to a session, allows more online time for debate and higher levels of learning. This could include evaluating new knowledge with respect to a challenging proton therapy plan and creating solutions with educators and learners.

## The future

In the post-pandemic era, the benefits of online learning will undoubtedly overshadow our pre-conceived biases, and will shape the future of proton therapy education. Online learning will not be a pandemic plug, but will be here to stay. Hybrid face-to-face and online schools and conferences will be the next era of education in this field. A hybrid format could also provide better networking opportunities to those who feel this is important. To widen participation further, and encourage discussion or networking, it may be important to extend engagement to secure social media platforms. Learning outcomes from delegates, and further analysis into sessions found more or less useful by delegates, will take place to contribute towards an internationally accepted proton therapy education curriculum.

## Conclusions

The online Christie Proton School provides an effective framework for proton education for the future. Common concerns around learner engagement and frequent difficulty accessing online platforms were shown to be unfounded. The future will include an entirely online school as well as hybrid events, with some face-to-face learning. Asynchronous learning will be retained by recordings to aid accessibility for an international audience.
